# Dress syndrome secondaire à l’allopurinol

**DOI:** 10.11604/pamj.2018.30.120.15854

**Published:** 2018-06-12

**Authors:** Youssef Zemmez, Naoufal Hjira

**Affiliations:** 1 Service de Dermatologie, HMIMV Rabat, Maroc

**Keywords:** DRESS syndrome, allopurinol, corticothérapie, DRESS syndrome, allopurinol, corticosteroid therapy

## Abstract

We report the case of a 60-year old patient with a 1-month history of hyperuricemia treated with allopurinol. The patient presented to the Department of Dermatology with acute rash on the face and the lower limbs associated with fever, arthralgias and myalgias. Clinical examination showed symmetric macular erythema on the face at the level of the cheeks with discreet edema (A), erythematous plaques at the level of both legs with healthy skin areas extending progressively from the bottom-up. Lesions were very itchy with burning sensation (B). The examination of the oral cavity showed very painful erosive lesions at the level of the internal face of the cheeks. Lymph nodes were free. Paraclinical tests showed leukocyte counts at 20000, elevated transaminases > 100 IU, eosinophil counts at 1500, HHV6 serology was negative. The diagnosis of DRESS syndrome was retained. The patient underwent corticosteroid therapy at a dose of 1 mg/kg/day associated with symptomatic treatment. Treatment evolution was marked by spectacular improvement after 06 days (C and D) with regression of skin lesions and gradual normalization of laboratory results.

## Image en médecine

Patiente âgée de 60 ans, ayant comme antécédents pathologiques une hyper-uricémie depuis un mois traitée par Allopurinol, qui a consulté en dermatologie pour éruption cutanée aigue intéressant la face et les membres inférieurs dans un contexte de fièvre d’arthralgies et de myalgies. L’examen clinique a objectivé un érythème maculeux de la face siégeant au niveau des joues d’une façon symétrique avec un œdème discret (A), des placards érythémateux au niveau des 2 jambes avec espaces de peau saine s’étendant progressivement d’une façon ascendante, ces lésion étaient très prurigineuses avec une sensation de cuisson (B). L’examen de la cavité buccale a montré des lésions érosives au niveau de la face interne des joues très douloureuses. Les aires ganglionnaires étaient libres. Le bilan paraclinique a objectivé une hyperleucocytose à 20000, élévation des transaminases > 100 UI, éosinophilie à 1500, sérologie HHV6 était négative. Le diagnostic d’un DRESS Syndrome a été retenu. Une corticothérapie a été instaurée à une dose de 1 mg/Kg/jr associé à un traitement symptomatique. L’évolution était marquée par une amélioration spectaculaire après 06 jours de traitement (C et D) avec régression des lésions cutanées et normalisation progressive des paramètres biologiques.

**Figure 1:**
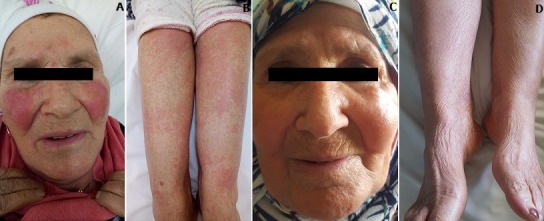
A) lésions érythémateuses avec oedème de la face; B) placards érythémateux symétrique des jambes; C) régression des lésions érythémateuses de la face; D) régression des placards érythémateux des jambes

